# Thyroid Hormone Receptor α Mutation Causes a Severe and Thyroxine-Resistant Skeletal Dysplasia in Female Mice

**DOI:** 10.1210/en.2013-2156

**Published:** 2014-06-10

**Authors:** J. H. Duncan Bassett, Alan Boyde, Tomas Zikmund, Holly Evans, Peter I. Croucher, Xuguang Zhu, Jeong Won Park, Sheue-yann Cheng, Graham R. Williams

**Affiliations:** Department of Medicine (J.H.D.B., G.R.W.), Imperial College London, London W12 0NN, United Kingdom; Dental Physical Sciences, Oral Growth and Development (A.B.), Queen Mary University of London, London E1 4NS, United Kingdom; Laboratory of X-Ray Micro-Computed Tomography and Nano-Computed Tomography (T.Z.), Central European Institute of Technology, Brno University of Technology CZ-61600 Brno, Czech Republic; Sheffield Myeloma Research Team (H.E.), University of Sheffield, Sheffield S10 2RX, United Kingdom; Bone Biology Program (P.I.C.), Garvan Institute of Medical Research, Sydney NSW 2010, Australia; and Laboratory of Molecular Biology (X.Z., J.W.P., S-y.C.), National Cancer Institute, Bethesda, Maryland 20892

## Abstract

A new genetic disorder has been identified that results from mutation of *THRA*, encoding thyroid hormone receptor α1 (TRα1). Affected children have a high serum T_3_:T_4_ ratio and variable degrees of intellectual deficit and constipation but exhibit a consistently severe skeletal dysplasia. In an attempt to improve developmental delay and alleviate symptoms of hypothyroidism, patients are receiving varying doses and durations of T_4_ treatment, but responses have been inconsistent so far. *Thra1*^*PV*/+^ mice express a similar potent dominant-negative mutant TRα1 to affected individuals, and thus represent an excellent disease model. We hypothesized that *Thra1*^*PV*/+^ mice could be used to predict the skeletal outcome of human *THRA* mutations and determine whether prolonged treatment with a supraphysiological dose of T_4_ ameliorates the skeletal abnormalities. Adult female *Thra1*^*PV*/+^ mice had short stature, grossly abnormal bone morphology but normal bone strength despite high bone mass. Although T_4_ treatment suppressed TSH secretion, it had no effect on skeletal maturation, linear growth, or bone mineralization, thus demonstrating profound tissue resistance to thyroid hormone. Despite this, prolonged T_4_ treatment abnormally increased bone stiffness and strength, suggesting the potential for detrimental consequences in the long term. Our studies establish that TRα1 has an essential role in the developing and adult skeleton and predict that patients with different *THRA* mutations will display variable responses to T_4_ treatment, which depend on the severity of the causative mutation.

The *THRA* and *THRB* genes encode the nuclear receptors (thyroid hormone receptor [TRα] and TRβ), which mediate thyroid hormone action in target tissues (1). Autosomal-dominant resistance to thyroid hormone (RTH) was recognized in 1967 (2), and the first causative mutations affecting *THRB* were identified 22 years later (3). More thanr 1000 RTH families have since been described, and affected individuals have increased thyroid hormone levels with an inappropriately normal or elevated TSH concentration due to disruption of the hypothalamus-pituitary-thyroid axis (4).

After the identification of *THRB* mutations in individuals with RTH it was a further 23 years before the first *THRA* mutations were reported in 2012 and 2013 (5–7). A six year-old girl with skeletal dysplasia and growth retardation was found to have a heterozygous *THRA* nonsense mutation resulting in expression of a truncated TRα1^E403X^ protein. She had normal serum TSH with low/normal T_4_ and high/normal T_3_ concentrations. Further investigations revealed macrocephaly with patent and abnormal skull sutures, delayed tooth eruption and bone age, disproportionate short stature, and epiphyseal dysgenesis with delayed mineralization of secondary ossification centers. Treatment with T_4_ for 9 months resulted in suppression of TSH and an increased basal metabolic rate but did not improve linear growth or skeletal development (5). A second girl with similar thyroid function and skeletal dysplasia was found to have a heterozygous frameshift mutation in *THRA* resulting in expression of a truncated TRα1^F397fs406X^ protein (6). She presented at the age of 3 years with macrocephaly, delayed tooth eruption, absent secondary ossification centers, and congenital hip dislocation. Reducing growth velocity became evident between 3 and 6 years of age. T_4_ treatment between 6 and 11 years of age only resulted in a small increase in growth velocity for a 2-month period but ultimately had no effect on her height, which continued along the 20th centile and was accompanied by persistently delayed bone age. The girl's 47-year-old father had the same *THRA* mutation and displayed short stature with a height 3.77 SDs below normal and acquired hearing loss due to otosclerosis (6, 8). Recently, a 45-year-old female with similar thyroid function, macrocephaly, and disproportionate short stature was identified and found to have a heterozygous frameshift mutation in *THRA*, resulting in expression of a truncated TRα1^P382fs388X^ protein. She presented in infancy with developmental delay and was treated intermittently with T_4_, which resulted in some improvement in growth velocity although her final adult height remained 2.34 SDs below normal (7).

These recent reports define a new genetic disorder characterized by a severe developmental phenotype with profound skeletal abnormalities that are thought to result from impaired T_3_ action in bone and cartilage (5–8). In an attempt to ameliorate the phenotype, three children have already received intermittent T_4_ at different doses and for varying durations. However, responses to date have been limited, and it is unknown whether long-term T_4_ treatment will be beneficial or detrimental. Thus, it is now essential to define the adult skeletal consequences of *THRA* mutations and determine the long-term effects of T_4_ supplementation, because life-long therapy is likely to be required. Importantly, Van Mullem et al (8) showed that dominant-negative inhibition of TRβ by TRα1^F397fs406X^ in vitro could be overcome partially by a high concentration of thyroid hormone. Furthermore, several studies have demonstrated that TRβ may mediate T_3_ actions in bone and cartilage (9–12), even though the principal physiological effects are mediated by TRα1. These observations suggest, therefore, that treatment of patients with supraphysiological doses of T_4_ may improve their skeletal abnormalities via TRβ-mediated actions. To address this timely question we investigated the effects of prolonged T_4_ treatment in a mouse model of this novel disease.

Mice with dominant-negative mutations affecting *Thra* (*Thra1^PV^*) and *Thrb* (*Thrb^PV^*) were generated to investigate the tissue-specific roles of TRα and TRβ and aid the identification of patients with *THRA* mutations (13, 14). The PV mutation, first recognized in a patient with RTH, is a C-insertion in exon 10 of *THRB* that results in a frameshift affecting the C-terminal 16 amino acids (15). The equivalent *Thra1^PV^* mutation comprises a homologous C-insertion followed by the PV sequence described in *THRB^PV^* (14, 16). The *Thra1^PV^* mutation disrupts helix 12 of TRα1, which is essential for T_3_ binding and coactivator recruitment (17) and lies within a 21-amino acid region containing the described human *THRA* mutations (5–7). Accordingly, TRα1^PV^ cannot bind T_3_ or activate target gene transcription but acts as a potent dominant-negative inhibitor of wild-type (WT) TRα1 or TRβ (14, 18, 19). Thus, the functional characteristics of TRα1^PV^ closely resemble those reported for TRα1^E403X^, TRα1^F397fs406X^, and TRα1^P382fs388X^ (5–7). Importantly, PV and none of the described human mutations affect the sequence of the TRα2 isoform that is also expressed from the *THRA* locus but which cannot bind T_3_ and has no known physiological function. Consistent with this, juvenile *Thra1*^*PV*/+^ mice display the same characteristics as children with heterozygous *THRA* mutations. They have a reduced T_4_:T_3_ ratio (14), delayed closure of the skull sutures with enlarged fontanelles, and severe postnatal growth retardation with delayed bone age. These abnormalities result from impaired TRα1-mediated T_3_ action in bone and cartilage (20–22), indicating that *Thra1^PV^* mice represent an excellent disease model in which to investigate the consequences of prolonged T_4_ treatment.

We hypothesized that the adult phenotype of *Thra1*^*PV*/+^ mice would predict the skeletal outcome of human *THRA* mutations and determine whether affected individuals may be susceptible to fracture or osteoarthritis, both of which are associated with altered thyroid hormone action in bone (23–26). We also hypothesized that prolonged treatment of *Thra1*^*PV*/+^ mice with a supraphysiological dose of T_4_ would ameliorate the developmental skeletal phenotype and improve bone structure and strength in adulthood.

The current studies demonstrate that adult *Thra1*^*PV*/+^ mice have short stature but normal bone strength despite high bone mass, suggesting that patients with *THRA* mutations are unlikely to have an increased risk of fracture. By contrast, gross morphologic abnormalities of the bones and joints predict that individuals with *THRA* mutations may be predisposed to osteoarthritis (27, 28). Although treatment with a supraphysiological dose of T_4_ completely suppressed TSH secretion, it had no effect on skeletal maturation, linear growth, or bone mineralization, thus demonstrating profound tissue resistance to thyroid hormone in *Thra1*^*PV*/+^ mice. However, prolonged T_4_ treatment increased bone stiffness and strength abnormally due to progressive enlargement of cortical bone diameter and thickness. Overall, the findings suggest that T_4_ treatment of individuals with dominant-negative *THRA* mutations is unlikely to improve their skeletal abnormalities substantially and may even be detrimental in the long term. Nevertheless, *Thra1*^*PV*/+^ mice represent an important disease model in which to identify and evaluate new therapeutic approaches.

## Materials and Methods

### *Thra1^PV^* mice

WT and heterozygous *Thra1*^*PV*/+^ mice have a mixed C57BL/6J and NIH Black Swiss genetic background and were bred and genotyped as described elsewhere (14, 21). Detailed characterization of the adult skeleton in *Thra1*^*PV*/+^ mice was performed in 14-week-old female mice after cessation of growth, and in fully mature 20-week-old female mice that had been treated with vehicle or T_4_ from weaning at 4 weeks of age until death. All mice were given ip injections of calcein (10 mg/kg in 100 μL PBS) 14 and 7 days before tissue collection (29).

### Ethics

Animal studies were performed according to the National Institutes of Health Guide for Care and Use of Laboratory Animals, and the National Cancer Institute Animal Care and Use Committee granted ethical approval for all experiments.

### Manipulation and measurement of thyroid status

TSH, T_4_, and T_3_ levels were determined in serum from mice (n = 5–13 per group) treated with vehicle or T_4_ (1.2 μg/mL in the drinking water) between 4–20 weeks of age. T_4_-supplemented water was changed every 3 days, with the T_4_ concentration adjusted to intake in 2-week cycles to ensure all animals received the same amount of T_4_ and did not become markedly thyrotoxic (14, 30–32).

### Histology

Tibias were fixed in 10% neutral buffered formalin and decalcified in 10% EDTA, embedded in paraffin wax. Sections (5 μm) were stained with alcian blue and van Gieson (29, 33). Measurements from at least 4 separate positions across the growth plate were obtained to calculate the mean height using a Leica DM LB2 microscope and DFC320 digital camera (Leica Microsystems). Results from 2 levels of sectioning were compared.

### Faxitron digital x-ray microradiography

Femurs were imaged at 10 μm resolution using a Faxitron MX20 (Qados). Bone mineral content was determined relative to steel, aluminum, and polyester standards. Images were calibrated with a digital micrometer, and bone length, cortical bone diameter, and thickness were determined (33, 34).

### Micro-computed tomography (CT)

Femurs were analyzed by micro-CT (Skyscan 1172a) at 50 kV and 200 μA with a detection pixel size of 4.3 μm^2^, and images were reconstructed using Skyscan NRecon software. A 1-mm^3^ region of interest was selected 0.2 mm from the growth plate, and trabecular bone volume as proportion of tissue volume (BV/TV), trabecular number, and trabecular thickness were determined (29, 33). Representative femurs from each treatment group were rescanned using a SCANCO μCT 40 (SCANCO Medical AG) operating at 55 kVp peak energy detection, 6 μm resolution to obtain approximately 2500 cross-sections per specimen in 766 × 763 pixel 16 bit DICOM files. Raw data were imported using 32-bit Drishti v2.0.221 (Australian National University Supercomputer Facility, http://anusf.anu.edu.au/Vizlab/drishti/) and rendered using 64-bit Drishti v2.0.000 to generate high-resolution images.

### Back scattered electron-scanning electron microscopy (EM) (BSE-SEM)

Femurs were fixed in 70% ethanol and opened longitudinally (33). Carbon-coated samples were imaged using backscattered electrons with a Zeiss DSM962 digital scanning electron microscope (EM) at 20-kV beam potential (KE Electronics). High-resolution images were quantified using ImageJ to determine the fraction of trabecular and endosteal bone surfaces displaying osteoclastic resorption (33).

### Quantitative BSE-SEM

Bone mineralization was determined by quantitative BSE-SEM at 1-μm^3^ resolution. Specimens were embedded in methacrylate and block faces polished to an optical finish for scanning electron microscopy (EM) analysis at 20 kV, 0.5nA with a working distance of 11 mm (33). Gradations of micromineralization density were represented in 8 equal intervals by a pseudocolor scheme (33, 35).

### Osteoclasts

Sections from decalcified tibias were stained for tartrate-resistant acid phosphatase, counterstained with aniline blue, and imaged using a Leica DM LB2 microscope and DFC320 digital camera (29, 33). A montage of 9 overlapping fields covering an area of 1 mm^2^ located 0.2 mm below the growth plate was constructed for each bone. BV/TV was measured, and osteoclast numbers and surface were determined in trabecular bone normalized to total bone surface (BS) (29, 33).

### Osteoblasts

Methacrylate-embedded specimens were imaged with a Leica SP2 reflection confocal microscope at 488-nm excitation to determine the fraction of BS undergoing active bone formation (33, 36). Mineral apposition rate was calculated by determining the separation between calcein labels at 20 locations per specimen beginning 0.2 mm below the growth plate. BS and mineralizing surface were measured using ImageJ, and the bone formation rate was calculated by multiplying mineralizing surface and mineral apposition rate.

### Bone strength

Three-point bend tests were performed on tibias, with a constant rate of displacement of 0.03 mm/s until fracture, using an Instron 5543 load frame and 100N load cell (Instron Limited). Biomechanical variables reflecting cortical bone strength were derived from load displacement curves (33, 37).

### Statistics

Data were analyzed by unpaired two-tailed Student's t test; *P* < .05 was considered significant. Frequency distributions of mineralization densities obtained by Faxitron and quantitative BSE were compared using the Kolmogorov-Smirnov test (29, 33, 34).

## Results

### Thyroid status and response to T_4_ administration in Thra1^PV/+^ mice

**Figure 1.**
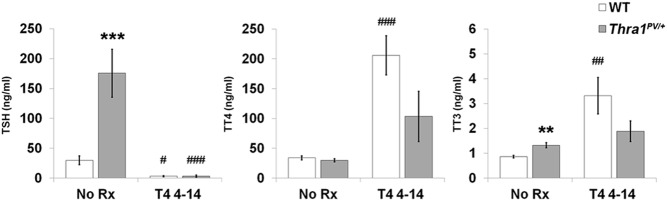
Thyroid status and response to T_4_ administration in *Thra1*^*PV*/+^ mice. Serum TSH (ng/mL), total T_4_ (ng/mL), and total T_3_ (ng/mL) concentrations in 14-week-old WT and *Thra1*^*PV*/+^ mice (n = 5–13 per group) following treatment with vehicle (no Rx) or T_4_ between the ages of 4 and 14 weeks. Statistical comparisons: 1) WT vs *Thra1*^*PV*/+^, Student's *t* test, **, *P* < .01, ***, *P* < .001; 2) no Rx vs T_4_ treatment, Student's *t* test, #, *P* < .05, ##, *P* < .01, ###, *P* < .001. Rx, treatment.

The thyroid status of adult WT and *Thra1*^*PV*/+^ mice was determined following treatment with vehicle or a supraphysiological dose of T_4_ from weaning until 14 weeks of age (Figure 1). The basal T_4_ concentration did not differ between WT and *Thra1*^*PV*/+^ mice, whereas T_3_ and TSH levels were increased in *Thra1*^*PV*/+^ mice by 1.5-fold (*P* < .01) and 6-fold (*P* < .001), respectively. Thus, the characteristically reduced T_4_:T_3_ ratio identified in individuals with *THRA* mutations (5–7) was also present in *Thra1*^*PV*/+^ mice (T_4_:T_3_ ratio: *Thra1*^*PV*/+^ 23 vs WT 39). Supraphysiological T_4_ treatment completely suppressed TSH in both WT and *Thra1*^*PV*/+^ mice. Despite profound and similar suppression of TSH, the increases in circulating T_4_ and T_3_ concentrations were attenuated in *Thra1*^*PV*/+^ mice (T_4_, 3.5-fold increase; T_3_, 1.5-fold) compared with WT (T_4_, 6-fold increase, *P* < .001; T_3_, 4-fold, *P* < .01) indicating that they are resistant to T_4_ administration.

### Delayed ossification and impaired bone modeling in Thra1^PV/+^ mice

**Figure 2.**
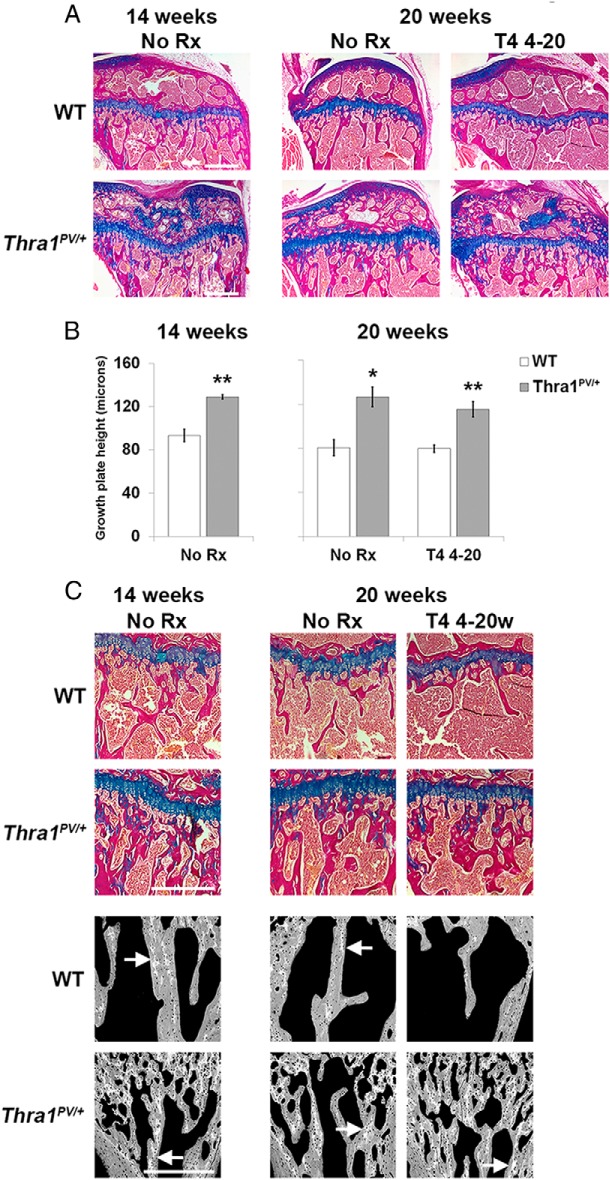
Effect of T_4_ treatment on the growth plate in *Thra1*^*PV*/+^ mice. A, Tibial growth plate sections stained with alcian blue (cartilage) and van Gieson (bone) from 14- and 20-week-old WT and *Thra1*^*PV*/+^ mice treated with vehicle (no Rx) or T_4_. Bar = 500 μm. B, Growth plate heights (mean ± SEM) in 14- and 20-week-old mice (n = 3 per genotype per group). Statistical comparisons: 14- and 20-week-old mice, WT vs *Thra1*^*PV*/+^, Student's *t* test, *, *P* < .05, **, *P* < .01. C, Panels show decalcified sections of tibial metaphysis stained with alcian blue and van Gieson from 14- and 20-week-old WT and *Thra1*^*PV*/+^ mice (bar = 500 μm) and undecalcified sections of caudal vertebrae imaged by quantitative BSE-SEM (bar = 500 μm). White arrows show increased amounts of highly mineralized cartilage retained within trabecular bone in *Thra1*^*PV*/+^ mice. Rx, treatment; 4–20w, 4–20 week.

Delayed bone development in juvenile *Thra1*^*PV*/+^ mice (21) led to severe skeletal abnormalities in adults. Growth plates in 14- and 20-week-old *Thra1*^*PV*/+^ mice were 39% and 70% wider than in WT mice (Figure 2, A and B), demonstrating persistent delay of endochondral ossification. An increased degree of retention of mineralized cartilage within trabeculae revealed that bone modeling was also impaired (Figure 2C). T_4_ administration did not affect either of these abnormalities in mutant mice (Figure 2 and data not shown).

### Structural consequences of defective ossification, modeling, and remodeling in adult Thra1^PV/+^ mice

**Figure 3.**
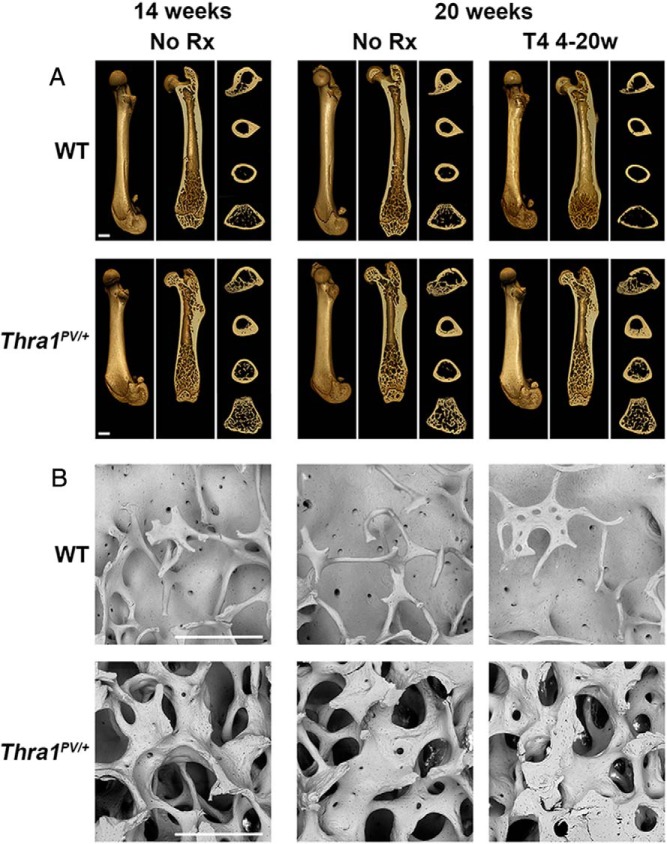
Effect of T_4_ treatment on bone structure in *Thra1*^*PV*/+^ mice. A, Micro-CT images of femurs from 14- and 20-week-old WT and *Thra1*^*PV*/+^ mice following treatment with vehicle (no Rx) or T_4_. A longitudinal image of the BS, a midline section, and transverse sections at 4 levels are shown. Bars = 1000 μm. B, BSE-SEM views of distal femur trabecular bone from 14- and 20-week-old WT and *Thra1*^*PV*/+^ mice. Bars = 500 μm. Rx, treatment; 4–20w, 4–20 week.

Bones from 14- and 20-week-old *Thra1*^*PV*/+^ mice were grossly dysmorphic. They were 17% and 15% shorter than WT and had splayed metaphyses, an abnormal cross-section throughout the diaphysis, and misshapen joint surfaces (Figure 3A). Micro-CT analysis indicated that trabecular bone volume, number, and thickness were increased in 20-week-old *Thra1*^*PV*/+^ mice (BV/TV, 2.1-fold; trabecular number, 1.9-fold; trabecular thickness, 1.1-fold greater) (Supplemental Figure 1), and these findings were confirmed by back-scattered electron-scanning EM (BSE-SEM) (Figure 3B). Similarly, cortical bone thickness (48% wider at 14 weeks, 43% at 20 weeks) and periosteal diameter (13% larger at 14 weeks, 20% at 20 weeks) were markedly increased in *Thra1*^*PV*/+^ mice (Supplemental Figure 1). T_4_ administration had no effect on these morphologic abnormalities (Figure 3A) but resulted in a gradual increase in cortical bone thickness and diameter in *Thra1*^*PV*/+^ mice (Supplemental Figure 1). Importantly, the endosteal diameter did not change in *Thra1*^*PV*/+^ mice following T_4_ treatment, whereas in WT mice it increased by 16% (*P* < .01). Thus, the increase in cortical bone thickness in *Thra1*^*PV*/+^ mice resulted from a failure of endosteal bone resorption combined with a likely increase in periosteal bone deposition.

### Increased bone mineral content but reduced mineralization in Thra1^PV/+^ mice

**Figure 4.**
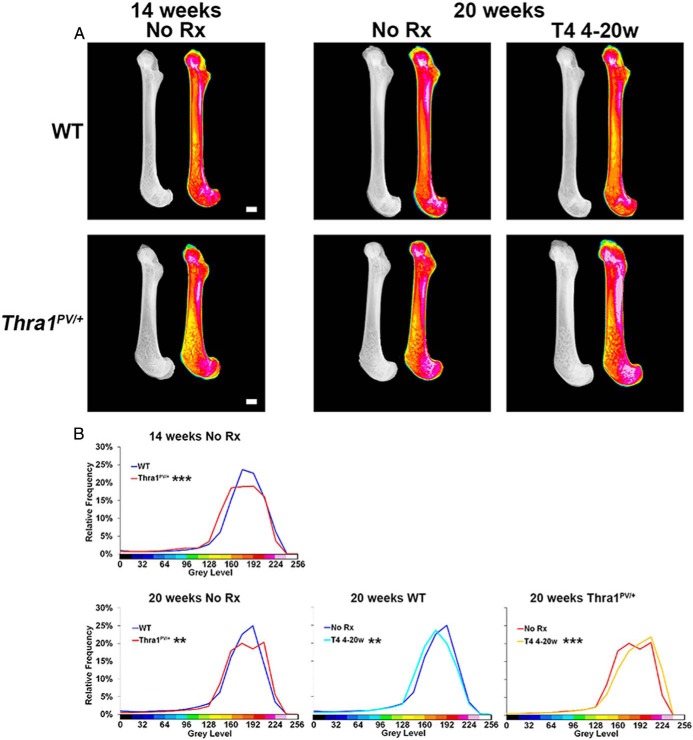
Effect of T_4_ treatment on bone mineral content in *Thra1*^*PV*/+^ mice. A, Quantitative Faxitron x-ray microradiography images of femurs from 14- and 20-week-old WT and *Thra1*^*PV*/+^ mice following treatment with vehicle (no Rx) or T_4_. Gray-scale images were pseudocolored according to a 16-color palette in which low mineral content is blue-black and high mineral content is pink-white. Bars = 1000 μm. B, Relative frequency histograms of femur bone mineral content (n = 3 per genotype per group). Kolmogorov-Smirnov test, WT vs *Thra1*^*PV*/+^ or no Rx vs T_4_ treatment, **, *P* < .01, ***, *P* < .001. Rx, treatment; 4–20w, 4–20 week.

**Figure 5.**
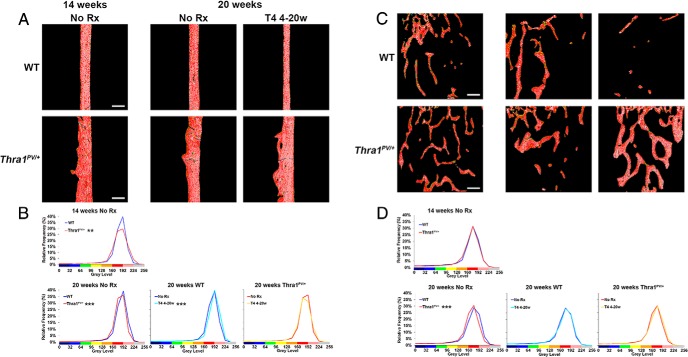
Effect of T_4_ treatment on bone mineralization density in *Thra1*^*PV*/+^ mice. A, Quantitative BSE-SEM images of femur mid-diaphysis cortical bone from 14- and 28-week-old WT and *Thra1*^*PV*/+^ mice following treatment with vehicle (no Rx) or T_4_. Gray-scale images were pseudocolored according to an 8-color palette in which low mineral content is blue and high mineral content is pink-gray. Bars = 200 μm. B, Relative frequency histograms of cortical bone micromineralization densities (n = 3 per genotype per group). C, Images of distal femur trabecular bone. Bars = 200 μm. D, Relative frequency histograms of trabecular bone micromineralization densities (n = 3 per genotype per group). Kolmogorov-Smirnov test, WT vs *Thra1*^*PV*/+^ or no Rx vs T_4_ treatment, **, *P* < .01, ***, *P* < .001. Rx, treatment; 4–20w, 4–20 week.

X-ray microradiography revealed that 14-week-old *Thra1*^*PV*/+^ mice had lower bone mineral content than WT mice, consistent with reduced mineral accrual during postnatal growth (21). Thus, in Figure 4A, the pseudocolored images in 14-week-old mice show more yellow and fewer red pixels in *Thra1*^*PV*/+^ mice compared with WT, indicating reduced bone mineral content. These differences are shown graphically in Figure 4B, in which the frequency distribution for *Thra1*^*PV*/+^ mice is shifted to the left. By contrast, in 20-week-old mice there was a small shift to the right in the pixel frequency distribution for *Thra1*^*PV*/+^, mice indicating higher, rather than lower, bone mineral content in older animals (Figure 4, A and B). Remarkably, supraphysiological T_4_ treatment further increased bone mineral content in *Thra1*^*PV*/+^ mice even though, as expected, it was reduced in WT mice following treatment (Figure 4, A and B). Thus, *Thra1*^*PV*/+^ mice were resistant to T_4_-induced bone loss and had a paradoxical increase in bone mineral content following treatment. Despite this, BSE-SEM revealed that cortical and trabecular bone mineralization density was reduced in 20 week-old *Thra1*^*PV*/+^ mice, the difference being greater in cortical bone, and that T_4_ treatment did not affect mineralization (Figure 5, A–D). Thus, *Thra1*^*PV*/+^ mice have an increase in bone mineral content (Figure 4) despite the reduction in tissue mineralization density (Figure 5) because their trabecular and cortical bone volume is substantially increased (Figure 2 and Supplemental Figure 1). Overall, therefore, *Thra1*^*PV*/+^ mice have increased cortical and trabecular bone volume compared with WT, but their bone is less mineralized.

### Reduced osteoclastic bone resorption in Thra1^PV/+^ mice

**Figure 6.**
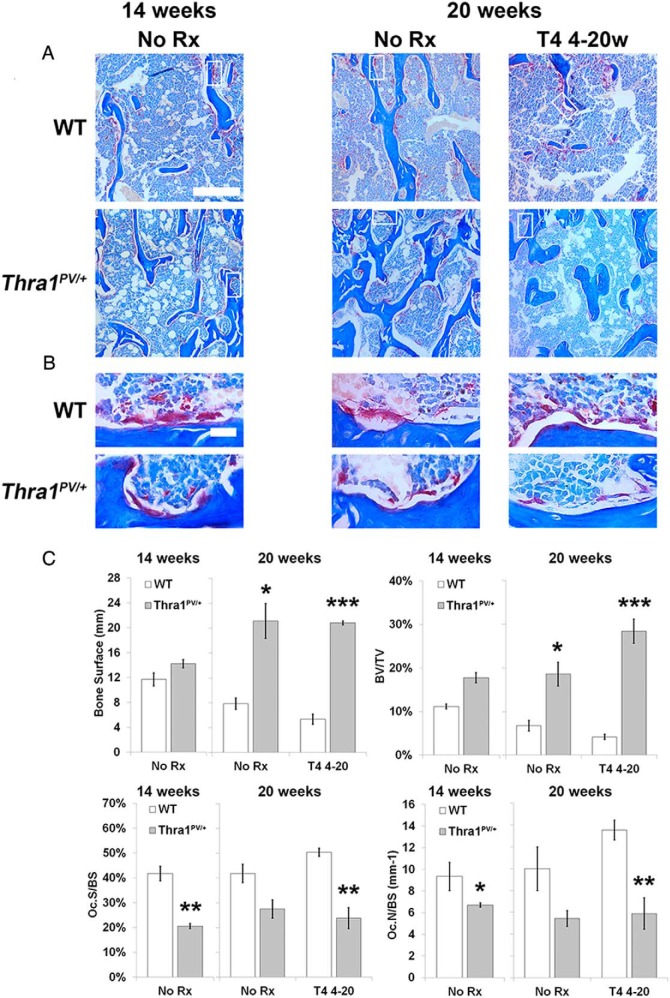
Effect of T_4_ treatment on osteoclastic bone resorption in *Thra1*^*PV*/+^ mice. A, Low-power views (bar = 100 μm) of tibia trabecular bone from 14- and 20-week-old WT and *Thra1*^*PV*/+^ mice following treatment with vehicle (no Rx) or T_4_, and stained for tartrate resistant acid phosphatase activity (pink) with aniline blue counterstain. The white boxes indicate the locations of the corresponding high-power images shown in panel B. B, High-power views (bar = 10 μm) of osteoclasts lining trabecular bone surfaces. C, Quantitative analysis of BS, BV/TV, osteoclast surface per bone surface (Oc.S/BS), and osteoclast number per BS (Oc.N/BS) (mean ± SEM) in 14- and 20-week-old mice (n = 3 per genotype per group). Statistical comparisons: 14- and 20-week-old mice, WT vs *Thra1*^*PV*/+^, Student's *t* test, *, *P* < .05, **, *P* < .01, ***, *P* < .001. Rx, treatment; 4–20w, 4–20 week.

Consistent with micro-CT and BSE-SEM analysis, histomorphometry studies demonstrated increased bone volume and surface in *Thra1*^*PV*/+^ mice. Furthermore, osteoclast surfaces were reduced and fewer osteoclasts were present in *Thra1*^*PV*/+^ mice compared with WT (Figure 6, A–C). Thus, *Thra1*^*PV*/+^ mice had a smaller proportion of their increased BS covered by osteoclasts (see also Supplemental Figure 2). The differences in BS, BV/TV, osteoclast surface/BS, and osteoclast number/BS between WT and *Thra1*^*PV*/+^ mice were accentuated following T_4_ treatment (Figure 6, A–C). Consistent with these findings, bone resorption was generally lower in *Thra1*^*PV*/+^ mice (Supplemental Figure 2) but bone formation parameters were similar (Supplemental Figure 3). However, it is important to note that small differences in dynamic bone formation may not have been detected in these studies because only 3 mice were analyzed per group.

### Abnormal bone stiffness and strength after prolonged T_4_ treatment of Thra1^PV/+^ mice

**Figure 7.**
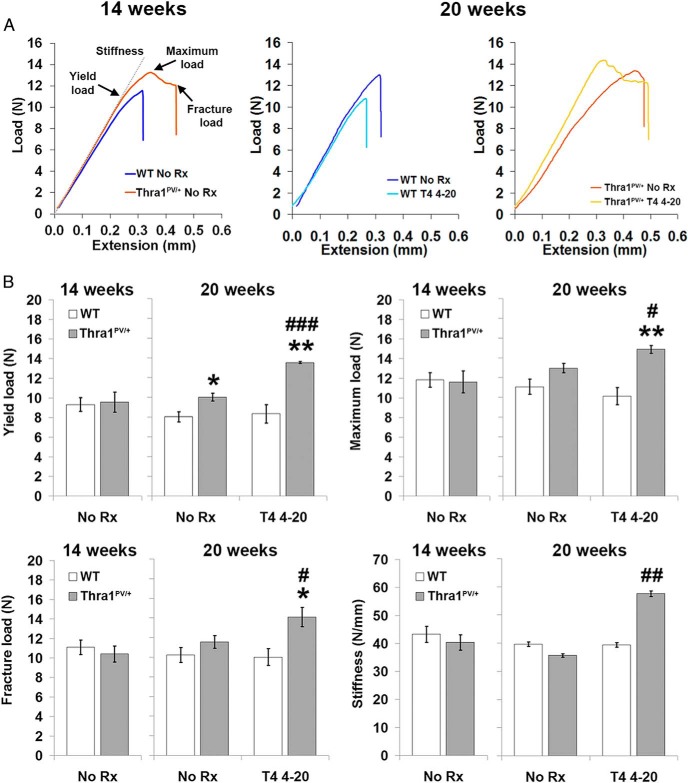
Effect of T_4_ treatment on cortical bone strength in *Thra1*^*PV*/+^ mice. A, Representative load-displacement curves from destructive 3-point bend testing of tibias from 14- and 20-week-old WT and *Thra1*^*PV*/+^ mice following treatment with vehicle (no Rx) or T_4_. B, Quantitative analysis of yield load, maximum load, fracture load, and stiffness (mean ± SEM) in 14- and 20-week-old mice (n = 3 per genotype per group). Statistical comparisons: 1) 14- and 20-week-old mice, WT vs *Thra1*^*PV*/+^, Student's *t* test, *, *P* < .05; 2) 20-week-old mice, no Rx vs T_4_ treatment from 4–20 weeks, Student's *t* test, #, *P* < .05, ##, *P* < .01, ###, *P* < .001. Rx, treatment.

Biomechanical testing revealed no difference in bone strength between untreated WT and *Thra1*^*PV*/+^ mice (Figure 7, A and B). Nevertheless, T_4_ treatment resulted in gradual increases in yield load, maximum load, fracture load, and stiffness of bones from *Thra1*^*PV*/+^ mice (Figure 7, A and B). Thus, prolonged T_4_ administration abnormally and progressively increased bone stiffness and strength in *Thra1*^*PV*/+^ mice.

## Discussion

### Skeletal phenotype resulting from mutation of Thra

During development *Thra1*^*PV*/+^ mice have delayed closure of the skull sutures, severe growth retardation, delayed bone age, and impaired bone mineral accrual (22). The delayed ossification persists into adulthood and is accompanied by impaired bone modeling and remodeling, resulting in short stature, increased bone mass, and gross morphologic abnormalities of the bones and joints, but normal bone strength. These findings suggest that, despite severe skeletal abnormalities, adults with *THRA* mutations are unlikely to have an increased risk of fracture. However morphologic abnormalities affecting the bones and joints predict that they may be at increased risk of osteoarthritis (27, 28).

### Cellular and molecular mechanisms

The abnormalities in *Thra1*^*PV*/+^ mice are consistent with effects of prolonged hypothyroidism on the growing and adult skeleton (38–42). Hypothyroidism disrupts growth plate chondrocyte differentiation leading to delayed endochondral ossification and linear growth, impairs bone modeling, and uncouples the processes of osteoclastic bone resorption and osteoblastic bone formation (43). In adults, even though it is well established that thyroid hormones increase bone resorption and promote bone loss, it is not known whether T_3_ acts directly in osteoclasts or whether effects on osteoclasts are secondary to the direct actions of T_3_ in osteoblasts (43). In *Thra1*^*PV*/+^ mice, prolonged impairment of chondrocyte differentiation is manifest by growth retardation and short stature in adulthood. Similarly, defective osteoclastic bone resorption is evidenced by reduced metaphyseal in-wasting, abnormal diaphyseal cross-section, and increased trabecular bone volume with retention of mineralized cartilage. Moreover, the grossly delayed formation of secondary ossification centers and reduced bone mineral accrual in *Thra1*^*PV*/+^ mice persisted throughout growth when mice were active and gaining weight. Thus, unmineralized epiphyses were exposed to abnormal and greater mechanical loads, resulting in compensatory enlargement of the epiphyses and metaphyses and culminating in adult joint deformity. Surprisingly, the strength of adult *Thra1*^*PV*/+^ bones was normal despite these structural abnormalities and is accounted for by the increased cortical bone thickness and diameter (33, 44).

A series of studies in genetically modified mice have shown that TRα1 is the principal mediator of T_3_ action in bone and cartilage (12, 21, 45–48). The finding of an identical skeletal phenotype in patients with *THRA* mutations (5–7) now demonstrates that TRα1 has a similar essential role in human bone development. Analysis of the mechanisms underlying the skeletal phenotypes in *Thra* mutant mice revealed decreased expression of T_3_ target genes including GH receptor (*Ghr*), insulin like growth factor-1 (*Igf1*), *Igf1* receptor (*Igf1r*), fibroblast growth factor receptor-1 (*Fgfr1*) and *Fgfr3*, and reduced downstream signaling responses mediated by the MAPK, signal transducer and activator of transcription 5, and AKT signaling pathways in chondrocytes and osteoblasts (12, 20, 21, 45, 49, 50). These data demonstrate impaired T_3_ action in cartilage and bone in *Thra* mutant mice despite a normal systemic T_3_ concentration and thus indicate the skeletal phenotype in individuals with *THRA* mutations is a consequence of local resistance to thyroid hormone.

The phenotypes in *Thra1*^*PV*/+^ mice and patients with *THRA* mutations result from the actions of potent dominant-negative mutant receptors. However, we have previously reported that mice harboring a less severe *Thra1^R384C^* mutation have a milder phenotype with only transiently delayed ossification and growth retardation, although modeling and remodeling defects resulting in increased bone mass, cortical thickness, and diameter were present in adults (45, 47). Importantly, and in contrast to *Thra1*^*PV*/+^ mice, treatment of *Thra1^R384C^* mice with a dose of T_3_ that overcomes the reduced ligand binding affinity and dominant-negative activity of the mutant receptor did ameliorate their skeletal abnormalities (45).

### Therapeutic approaches in individuals with THRA mutations

The response to thyroid hormone treatment in *Thra1^R384C^* mice suggests that individuals with *THRA* mutations may benefit from similar treatment. Unfortunately, however, doses of T_4_ sufficient to normalize circulating hormone concentrations have been largely ineffective in the patients treated so far (5–8), presumably because the currently identified individuals have mutations that result in expression of mutant receptors with little or no T_3_ binding affinity. Despite this, Van Mullem et al (8) showed that dominant-negative inhibition of TRβ by TRα1^F397fs406X^ in vitro could be overcome partially by increasing concentrations of thyroid hormones. In this context, several studies have suggested that TRβ can mediate T_3_ action in bone and cartilage (9–12), even though the principal physiological effects are mediated via TRα1. Thus, we hypothesized that treatment of *Thra1*^*PV*/+^ mice with a supraphysiological dose of T_4_ might improve bone structure and strength.

However, such treatment of *Thra1*^*PV*/+^ mice had no beneficial effect on growth or skeletal deformity but did, nevertheless, increase cortical bone thickness and diameter. These responses were likely mediated by TRβ and resulted in abnormal increases in bone stiffness and strength that may adversely affect the optimal compromise between strength and flexibility that is essential to minimize fracture risk (51). Thus, prolonged treatment of individuals harboring *THRA* mutations with high doses of T_4_ may also have adverse consequences in other tissues where T_3_ action is predominantly mediated via TRβ.

GH therapy represents an alternative approach to improve linear growth and skeletal maturation in children with *THRA* mutations, but treatment in one individual so far was ineffective (6). The reduced expression of *Ghr*, *Igf1*, and *Igf1r*, together with impaired signal transducer and activator of transcription 5 and AKT signaling in growth plate chondrocytes in *Thra* mutant mice (12, 21, 50), suggests a mechanism to account for this lack of clinical response to GH.

### Thyroid hormone metabolism and response to T_4_ administration in Thra1^PV/+^ mice

Thyroid hormone metabolism is mediated by 3 iodothyronine deiodinases. The type 1 enzyme (D1) catalyzes removal of an inner or outer ring iodine from T_4_ to generate T_3_ or rT_3_, or an outer ring iodine from rT_3_ to generate 3,3′-diiodothyronine. D1 is expressed in liver, kidney, and thyroid and contributes to the circulating concentration of T_3_ (52). The type 2 enzyme (D2) converts the prohormone T_4_ to the active hormone T_3_: it is expressed in the hypothalamus and pituitary and peripheral target tissues, where it generates a local supply of T_3_ and is subject to substrate-mediated inactivation (53). By contrast, the type 3 enzyme (D3) catalyzes removal of an inner ring iodine from T_4_ or T_3_ to generate the inactive metabolites rT_3_ or 3,3′-diiodothyronine. D3 expression is induced by thyroid hormone, thus limiting the supply of T_3_ in conditions of thyroid hormone excess (52).

Remarkably, and despite complete suppression of TSH, *Thra1*^*PV*/+^ mice had a blunted increase in circulating thyroid hormones following a supraphysiological dose of T_4_. This discrepancy indicates that the hypothalamus-pituitary-thyroid axis is intact in *Thra1*^*PV*/+^ mice, but metabolism of thyroid hormones must be increased. Indeed, we previously showed that untreated *Thra1*^*PV*/+^ mice have a 9-fold increase in hepatic D1 mRNA expression (14) resulting in a 4.8-fold increase in enzyme activity (54). It is well established that T_3_ acts via TRβ1 to stimulate D1 expression in the liver (55, 56) and, accordingly, hepatic D1 activity is increased further in *Thra1*^*PV*/+^ mice following treatment with T_3_ (54, 57). By contrast, T_3_ acts via TRα1 to stimulate expression of D3 (58) and we previously demonstrated that T_3_ treatment of *Thra1*^*PV*/+^ mice fails to induce the normal increase in D3 activity observed in WT animals (54, 57).

**Figure 8.**
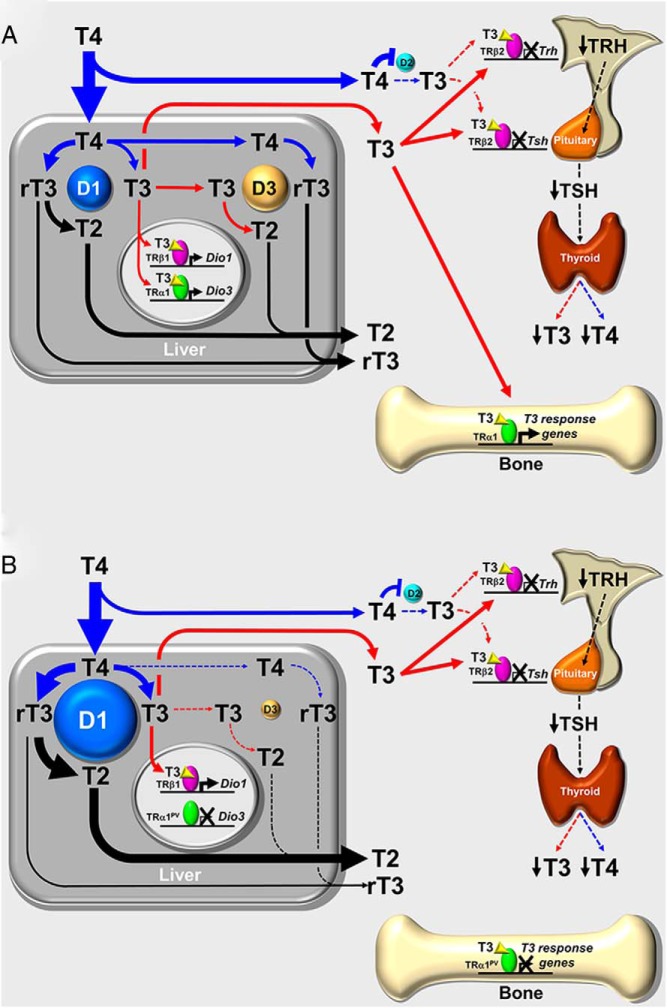
Proposed model for attenuated systemic response to T_4_ administration in *Thra1*^*PV*/+^ mice. A, Normal response in WT mice. High concentrations of T_4_ are metabolized in the liver. D1 converts T_4_ to rT_3_ or T_3_, and rT3 is metabolized to 3,3′-diiodothyronine (T_2_). Acting via TRβ1, T_3_ increases D1 expression to complete a feed-forward loop. However, T_3_ also acts via TRα1 to increase D3 expression and thus limit feed-forward activation of D1. Thus, T_4_ excess results in a parallel increase in both D1 and D3 so that levels of T_3_, rT_3_, and T_2_ in the circulation rise to reflect increased T_4_ metabolism. The high levels of circulating thyroid hormones suppress TRH and TSH expression and inhibit endogenous T_4_ and T_3_ production. At steady state, most circulating T_3_ is derived from increased D1-mediated metabolism of T_4_. The TRα1-mediated actions of T_3_ in bone are increased. B, Abnormal response in *Thra1*^*PV*/+^ mice. High concentrations of T_4_ are metabolized in the liver. D1 converts T_4_ to rT_3_ or T_3_, and rT_3_ is metabolized to T_2_. Acting via TRβ1, T_3_ increases D1 expression to complete a feed-forward loop. However, in *Thra1*^*PV*/+^ mice the mutant TRα1^PV^ prevents T_3_ stimulation of D3 expression, thus maintaining feed-forward activation of D1. Administration of T_4_ fuels this feed-forward activation and would result in enhanced metabolism of T_4_, and ultimately increased accumulation of T_2_. Thus, although circulating T_3_ and T_4_ levels rise to a lesser degree than in WT animals, they are still sufficient to suppress the hypothalamus-pituitary-thyroid axis. At steady state, the grossly increased D1 activity thus accounts for resistance of *Thra1*^*PV*/+^ mice to T_4_ administration. Despite exogenous thyroid hormone administration, T_3_ action in bone remains inhibited by dominant-negative TRα1^PV^ (21).

We propose, therefore, that the resistance to T_4_ administration observed in *Thra1*^*PV*/+^ mice results from the markedly increased D1 activity combined with this absent D3 response (Figure 8). Consistent with this model, TSH in individuals with *THRA* mutations was suppressed readily following T_4_ treatment despite only small increases in T_4_ and T_3_ concentrations (7, 8). Detailed future metabolic studies will be required to confirm the precise underlying mechanisms responsible for these findings. For example, because defects in TRα1 action may result in intestinal problems, it is possible that absorption of orally administered T_4_ could be impaired in *Thra1*^*PV*/+^ mice. However, it should also be noted that, following oral treatment with T_4_, the TSH concentration was suppressed completely in both WT and *Thra1*^*PV*/+^ mice, indicating that intestinal absorption of T_4_ was unlikely to be markedly impaired in *Thra1*^*PV*/+^ mice. Nevertheless, it would be instructive to investigate whether differences in serum T_4_ and T_3_ levels persist between WT and *Thra1*^*PV*/+^ mice following parenteral administration of T_4_.

## Conclusions

The overall resistance of the skeleton to T_4_ treatment in *Thra1*^*PV*/+^ mice and the patients studied so far is likely to be a consequence of the potent dominant-negative activities of their mutant TRα1 proteins (5–7, 14, 18). It is inevitable, however, that individuals with less severe *THRA* mutations will be identified in the future and, in such cases, T_4_ treatment is likely to be beneficial. Thus, treatment of *Thra1^R384C^* mice with doses of T_4_ that overcome the reduced binding affinity of TRα1^R384C^ rescued their skeletal phenotype by preventing delayed ossification and growth retardation, ultimately ameliorating adult bone structure and mineralization (45). Taken together, these studies predict that individuals with *THRA* mutations will display variable degrees of skeletal deformity and different responses to T_4_ treatment that correlate with the functional consequences of the particular disease-causative mutation. Therefore, in patients with *THRA* mutations, it will be important to characterize the functional properties of their mutant TRα1 because this may predict their response to T_4_ treatment and the optimal systemic T_4_ concentration required.
